# Interplay Effect of Temperature and Excitation Intensity on the Photoluminescence Characteristics of InGaAs/GaAs Surface Quantum Dots

**DOI:** 10.1186/s11671-018-2792-y

**Published:** 2018-11-29

**Authors:** Qing Yuan, Baolai Liang, Chuan Zhou, Ying Wang, Yingnan Guo, Shufang Wang, Guangsheng Fu, Yuriy I. Mazur, Morgan E. Ware, Gregory J. Salamo

**Affiliations:** 1 Hebei Key Laboratory of Optic-electronic Information and Materials, College of Physics Science & Technology, Hebei University, Baoding, 071002 People’s Republic of China; 2 Institute for Nanoscience and Engineering, University of Arkansas, Fayetteville, AR 72701 USA

**Keywords:** Surface quantum dots, Photoluminescence, Carrier dynamics, Interaction

## Abstract

We investigate the optical properties of InGaAs surface quantum dots (SQDs) in a composite nanostructure with a layer of similarly grown buried quantum dots (BQDs) separated by a thick GaAs spacer, but with varied areal densities of SQDs controlled by using different growth temperatures. Such SQDs behave differently from the BQDs, depending on the surface morphology. Dedicated photoluminescence (PL) measurements for the SQDs grown at 505 °C reveal that the SQD emission follows different relaxation channels while exhibiting abnormal thermal quenching. The PL intensity ratio between the SQDs and BQDs demonstrates interplay between excitation intensity and temperature. These observations suggest a strong dependence on the surface for carrier dynamics of the SQDs, depending on the temperature and excitation intensity.

## Introduction

Self-assembled In(Ga)As/GaAs semiconductor quantum dots (QDs) have attracted extensive research interest since 1992 due to their unique physical properties and their wide range of potential applications [1, 2]. Generally, self-assembled In(Ga)As semiconductor QDs are grown on GaAs substrates and are subsequently buried (buried QDs, or BQDs) in a GaAs matrix to confine the wave function of carriers inside the QDs in all dimensions with stable barriers resulting from the GaAs to In(Ga)As band offsets. Such In(Ga)As/GaAs BQDs have been widely applied as the active region materials for many devices like lasers, detectors, modulators, photovoltaic, memory cells, and so on [3–7].

When the In(Ga)As QDs are left on the GaAs surface (surface QDs, or SQDs) without a GaAs capping layer and directly exposed to air, the confinement of the wave function in the growth direction is sensitively coupled to the chemical composition of the air and the surrounding environment. As a result, their optical and electronic behaviors become very sensitive to fluctuations in that environment [8–11]. Such surface-sensitive properties indicate that SQD structures could gain an important role in sensor applications [12–15]. For example, high sensitivity humidity sensors based on self-assembled InGaAs SQDs have been proposed [16].

In order to realize such surface-sensitive detection systems, it is necessary to explore the underlying physical mechanisms which govern the optical and transport performance in these In(Ga)As SQD structures. Previously, we have studied a hybrid structure with InGaAs SQDs and revealed a carrier transfer process between the surface states and the SQDs through photoluminescence (PL) measurement [17]. In this work, we further investigate the optical performance of composite nanostructures with the InGaAs SQDs separated from an InGaAs BQD layer by a thick GaAs spacer, but with varied SQD surface densities controlled by using different growth temperatures. Such SQDs behave differently from the BQDs, depending on the surface morphology. In particular, PL spectra of the SQDs grown at 505 °C are carefully studied with respect to excitation intensity and temperature. The results indicate that the interaction between surface states and SQDs strongly depends on the temperature and excitation intensity.

## Methods

**Fig. 1:**
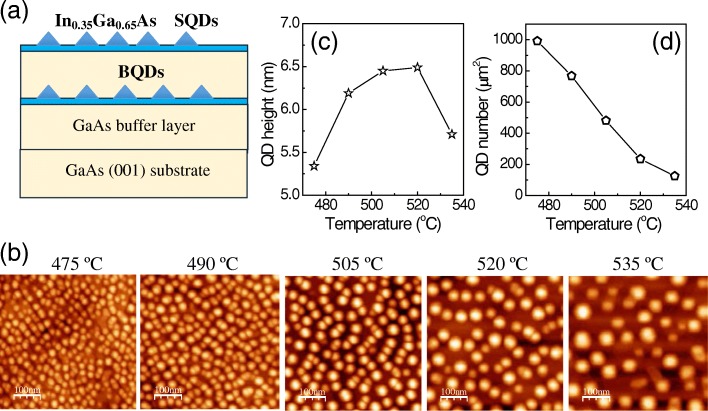
**a** The schematic diagrams of the SQD sample structure. **b** 0.5 μm × 0.5 μm AFM images of the InGaAs SQDs grown at different temperatures. **c** The average height and **d** areal density of the InGaAs SQDs are plotted with respect to the growth temperature

Five samples were grown on the GaAs (001) semi-insulating substrates by a solid-source VEECO Gen-930 molecular beam epitaxy (MBE). As shown in Fig. 1a, after the oxide layer desorption and the growth of a 200 nm GaAs buffer at 580 °C, the substrate temperature was decreased to 475 °C, 490 °C, 505 °C, 525 °C, or 535 °C, respectively, where 11 monolayers (MLs) of In_0.35_Ga_0.65_As were deposited to form the BQD layer. This was followed by 70 nm of GaAs and another 11 MLs of In_0.35_Ga_0.65_As grown at the same temperature to form the SQDs. Finally, the sample was cooled under arsenic flux to 300 °C and taken out of the MBE chamber. Upon removal from the MBE and between experiments, the samples were stored in a dry nitrogen-gas cabinet at room temperature.

The In_0.35_Ga_0.65_As SQDs were studied for each sample by atomic force microscopy (AFM) using tapping mode in air at room temperature. For PL measurements, the samples were loaded into a closed-cycle JANIS CCS-150 optical cryostat with a vacuum of < 10^−5^ Torr and variable temperature (10–300 K). The QD samples were excited by a solid-state 532 nm laser through a × 20 infinity-corrected objective lens. The PL signal was collected by the same objective lens and focused onto the entrance slit of a 0.5-m Acton-2500 spectrometer and subsequently detected by a liquid nitrogen-cooled Princeton Instruments PyLoN-IR CCD detector.

## Results and Discussion

The morphology of the In_0.35_Ga_0.65_As SQDs is studied for each sample, as indicated by the AFM images in Fig. 1b and the extracted QD height in Fig. 1c as well as the QD density in Fig. 1d. For all samples, large incoherent islands or defects are not found on the surface as expected for high-quality QD samples. For growth temperatures increasing from 475 to 535 °C, we find the areal density of the SQDs to monotonically decrease from 9.86 × 10^10^ to 1.25 × 10^10^ cm^−2^. Such QD density change is due to the enhancement of adatom diffusion length with increasing substrate temperature. Interestingly, the average height of SQDs does not monotonically depend on the growth temperature. It reaches a maximum of 6.5 nm for the sample grown at 520 °C, indicating an indium-desorption effect at higher growth temperature.

**Fig. 2:**
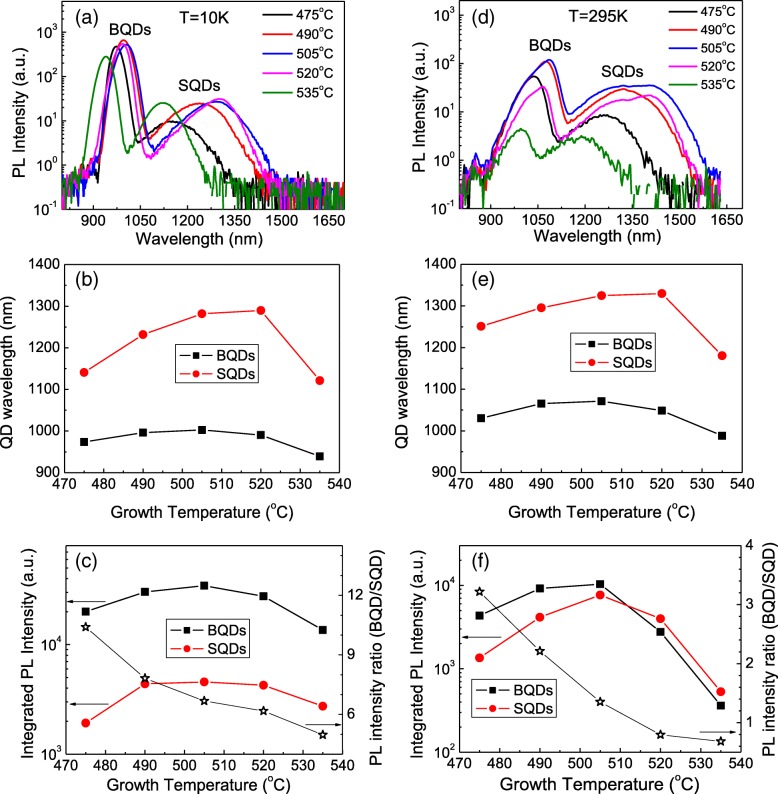
**a** PL spectra measured at 10 K with an excitation laser intensity of 20 W/cm^2^. **b** Extracted PL wavelength and **c** integrated PL intensity as a function of the growth temperature. **d** PL spectra measured at 295 K with an excitation laser intensity of 200 W/cm^2^. **e** PL wavelength and **f** integrated PL intensity as functions of the growth temperature

The PL spectra were first measured with a relatively low excitation intensity of 20 W/cm^2^ at 10 K. As shown in Fig. 2a–c, the spectra show two obvious bands of emission for each sample. The long wavelength emission is attributed to the SQDs with the shorter wavelength peak being from the BQDs. Here, we find distinct features of the PL wavelengths, full width at half maximums (FWHMs), and intensities between the SQDs and BQDs. The red-shift for SQD emission with respect to BQD emission is attributed to the changes of strain, QD dimension, and indium intermixing before and after growing the GaAs capping layer, i.e., the BQDs are under a greater compressive strain, smaller average QD height, and stronger intermixing with a commensurate band-gap shift to higher energies [18–20]. The large FWHM of the SQDs is likely due to the coupling between the surface states and the confined energy states in the QDs. In consideration of the PL intensity, it can be seen that the BQDs always have the emission intensity much stronger than the SQDs and the integrated PL intensity ratio varies with respect to the samples grown at different temperatures. The samples grown at 505 °C present the maximum intensity for both BQDs and SQDs, indicating the best QD quality for this sample.

PL spectra were then measured with an excitation intensity of 200 W/cm^2^ at room temperature. As shown in Figs. 2d–f, both the SQD peak and the BQD peak move to longer wavelengths with increasing temperature from 10 K to 295 K. Both the wavelength and the integrated PL intensity follow similar behavior as they did at 10 K. But, very interestingly, we find the ratio of the PL intensities of the BQDs to the SQDs integrated over the full width of each band is significantly different at low temperatures than it is at 295 K, for example, for the sample grown at 505 °C it is ~ 6.7 at 10 K, whereas it is ~ 1.35 at room temperature. This indicates that the SQDs and BQDs have different carrier recombination characteristics and underlying mechanisms for PL quenching, depending on the SQD density, the temperature, and probably the excitation intensity (i.e., the carrier population in the QDs). It is the surface states that can act as nonradiative centers and “freeze” photon-generated carriers at low temperature. But these confined carriers can be thermally activated at high temperature to enhance SQD emission [17]. We select the sample grown at 505 °C to do a more dedicated excitation-dependent and temperature-dependent PL investigation as it shows the best QD quality for both SQDs and BQDs.

**Fig. 3:**
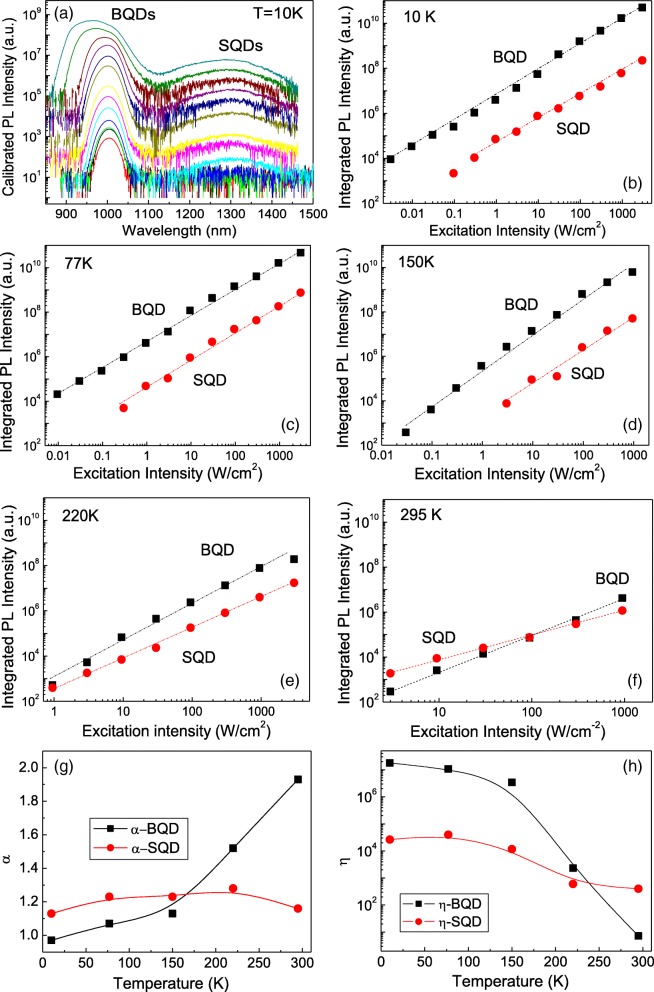
**a** PL spectra as a function of the excitation intensity for the sample grown at 505 °C. **b**~**f** The integrated PL intensities of the BQDs and SQDs as functions of the excitation intensity at 10 K, 77 K, 150 K, 220 K, and 295 K respectively. **g**, **h** The power law parameters *α* and *η* for BQDs and SQDs at different temperature. Here, the lines are only guides-to-the-eye

For the sample grown at 505 °C, the PL spectra are then measured for both SQDs and BQDs as a function of the excitation laser intensity at temperatures of 10 K, 77 K, 150 K, 220 K, and 295 K. Figure 3a shows the measured spectra at 10 K as an example. From the excitation intensity-dependent PL spectra, the integrated PL intensity is extracted as a function of the excitation laser intensity at each temperature. As shown in Fig. 3b–f, the PL intensities increase linearly with increasing excitation intensity. A generalized power law of *I*_PL_ = *η* × *P*^*α*^ is satisfied in the low excitation range, where *P* is the power density of the excitation laser and *I*_PL_ is the integrated intensity of the QD emission. The exponent *α*, depending on the radiative recombination mechanisms, is expected to be close to unity for exciton recombination and 2 for free carrier recombination. The coefficient *η* is actually a comprehensive characteristic that includes absorption, capture, and recombination of excitons [21, 22]. The exponents, *α*, and coefficients, *η*, are plotted in Fig. 3g, h, respectively. They are obtained by fitting the experimental data in Fig. 3b–f for the five measured temperatures, 10 K, 77 K, 150 K, 220 K, and 295 K, respectively. Different temperature-dependencies can be seen for BQDs and SQDs.

For the exponent *α*, we find that it is in-fact unity at low temperatures between 10 and 150 K for the BQDs but it increases to 1.9 with increasing temperature from 150 to 295 K. This indicates exciton recombination for the BQDs in the low-temperature regime but a more complicated carrier recombination mechanism for higher temperatures. For pure exciton recombination, the coefficient, *α*, should be smaller than unity, because the increase in excitation intensity increases the optical dissipation as a result of the increased light diffusion and nonradiative carrier losses [21]. For the SQDs, however, *α* is observably larger (*α* = 1.2~1.3) than unity with very little variation over the entire temperature range, from 10 to 295 K. Therefore, the SQD emission at low temperature is not purely exciton-like. It may already include nonradiative recombination mechanisms at levels greater than the BQDs.

The coefficient *η* can be seen to slowly decrease with increasing temperature from 10 to 150 K for the BQDs, then quickly decrease from 150 to 295 K. However, for the SQDs, *η* slowly decreases throughout the entire temperature range from 10 to 295 K. We also find that *η* for the BQDs is almost two orders larger than that for the SQDs at low temperatures from 10 to 150 K, indicating weak PL efficiency for the SQDs at such low temperatures. However, at 150 K *η* for the BQDs begins to dramatically decrease with increasing temperature becoming nearly two orders of magnitude less than that of the SQDs at room temperature.

The observed behaviors of the exponent *α* and the coefficient *η* in Fig. 3g, h clearly reinforce our assumption that the SQDs and BQDs have different features and underlying mechanisms for emission and PL quenching. For the BQDs, the carriers are confined inside the QDs at low temperature of 10 K and emission by exciton recombination is dominant. With increasing temperature from 10 to 77 K and then to 150 K, the carriers gain energy from phonons which enables them to be activated from small dots and redistribute into larger ones. With increasing the temperature further from 150 K toward room temperature, the carriers gain enough energy to escape from the BQDs to nonradiative centers, resulting in thermal quenching of the PL signal. Therefore, the BQDs have no direct interaction with the surface states. It is phonons that make the carriers inside the BQDs redistribute and quench.

In contrast, the SQDs are intimately contacted with the surface states [17, 20]. At low temperature, there is strong competition between SQDs and surface states for receiving photon generated carriers from the GaAs matrix. Clearly, due to the high density of the surface states, they receive more carriers than the SQDs do. As a result, we observed weak PL intensity for the SQDs at 10 K. Also, due to the coupling or cross-talk between SQDs and surface states, the exponent α, is observably larger (*α* = 1.2~ 1.3) than unity for SQDs at 10 K. With increasing temperature, the carriers confined in the surface states may gain phonon energy to escape and then to populate the SQDs [17]. This recapture of carriers enhances the emission from the SQDs and not the BQDs at high temperature. This explains the slight increase of the coefficient, *η*, while the temperature increases from 10 to 77 K as is shown in Fig. 3h. This also explains why the value of the coefficient, *η*, from the SQDs becomes higher than that from the BQDs at ~ 220 K in the same figure. Above all, we observe that the SQD emission did not vary as much as the BQDs did with temperature with regards to the coefficient, *η*, and exponent, *α*. Thus, the carrier dynamic process shows different temperature dependences for BQDs and SQDs.

**Fig. 4:**
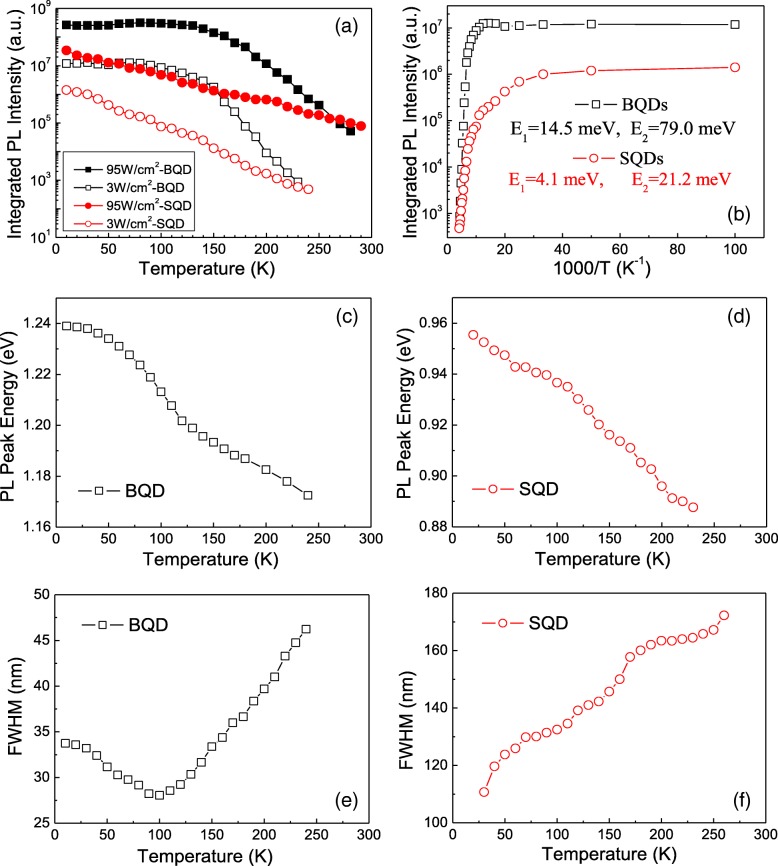
**a** Integrated PL intensities of the BQDs and the SQDs as functions of temperature at different excitation intensities. **b** Arrhenius plot with an excitation intensity 3 W/cm^2^ for the BQDs and the SQDs. The PL peak energy of **c** the BQDs and **d** the SQDs. The FWHM of **e** the BQDs and **f** the SQDs as functions of temperature

To further characterize the SQDs, temperature-dependent PL spectra was measured at different excitation intensities. This is shown in Fig. 4. Here, again we find different characteristics between the SQDs and the BQDs. For the BQDs, in Fig. 4a, the evolution of the integrated PL intensity as a function of temperature shows two regimes. For each excitation intensity, the integrated PL intensity remains constant until some critical temperature, above which it decays rapidly. This is a typical behavior for PL from InGaAs BQDs. In the low-temperature regime, some carriers can gain thermal energy to be activated and recaptured by larger BQDs. Therefore, in this regime, there is no prominent loss in the integrated PL intensity, but the PL peak energy is found to decrease as the FWHM becomes narrower, as shown in Fig. 4c, e. In the high-temperature regime, carriers in BQDs gain enough thermal energy to escape from the BQDs and subsequently get trapped on nonradiative carrier traps, which makes the integrated PL intensity decay due to loss of carriers from the BQDs. The two regimes seen here for the BQDs in Fig. 4 correlate with the variations in the exponent, *α* and coefficient, *η* for SQDs as shown in Fig. 3g, h, reflecting the same mechanisms in the temperature-dependent PL measurements.

For the SQDs, in Fig. 4a, the integrated PL intensity monotonically decreases throughout the entire range of the measured temperatures. We observe that the integrated PL intensity of the SQDs decreases faster/slower than that of the BQDs in the low-/high-temperature regime with a turn-over at ~ 150 K. Interestingly, the SQDs did not show carrier recapture features in the low-temperature regime of 10 K ~ 80 K as was observed previously [17]. This is most likely due to differences in the QD density and/or the excitation intensity. We also observe in Fig. 4a that the integrated PL intensity of the SQDs begins to decrease as soon as the temperature increases from 10 K. Some groups have attributed the earlier thermal quenching of the SQD PL intensity to the sensitivity of the SQDs to environmental potential fluctuations [23, 24]. Others claim that there are no confined electron states in the wetting layer of InGaAs SQDs so that the carriers confined in SQDs lack a channel to transfer to other larger SQDs through thermal activation and recapture [17, 20].

Here, we present a different hypothesis to explain the thermal quenching of the SQD PL. We believe that the surface states play an important role for the SQD emission and quenching. The surface states strongly couple with the discrete energy states of the SQDs, which allow for carriers to easily transfer to nonradiative traps even at low temperature. Therefore, the integrated PL intensity of the SQDs decreases faster than that of BQDs in the low-temperature regime. In the high-temperature regime where the BQDs begin to quench quickly due to carrier escape to the WL and the GaAs, we see that the SQD quenching is slower than the BQDs. This is like the combined result of two properties of the system. First, the SQDs have deeper confined electron energy levels than the BQDs as indicated by their lower energy PL. Second, there are no confined electron states in the wetting layer of the InGaAs SQDs, and therefore the carriers confined in the SQDs lack an efficient channel to transfer to other larger SQDs through thermal activation and recapture. This is only possible through the surface state channel. This continues to draw carriers out of the SQDs at the same rate; therefore, there is no sudden quenching like for the BQDs. In addition, the carrier transfer from the surface states to the SQDs would also enhance the SQD emission.

Through temperature-dependent PL measurements, we have observed that the SQDs begin to quench at lower temperature but ultimately their intensity decreases slower than the BQDs do at high temperature. In addition, we find that the higher the excitation intensity, the slower the thermal decay rate of the integrated PL intensity for SQDs. It is reasonable to assume that, at higher excitation intensity, the surface states become more populated, thus reducing the loss of carriers from the SQDs. Subsequently, the integrated PL intensity of the SQDs exhibits a more gradual thermal decay with increasing excitation intensity.

In order to better understand the mechanism of carrier thermal quenching, Fig. 4b shows an Arrhenius plot with an excitation intensity of 3 W/cm^2^. The experimental data was fitted with a relation involving two nonradiative recombination processes:

$$ I(T)=\alpha /\left[1+{C}_1\exp \left(-{E}_1/\left({k}_BT\right)\right)+{C}_2\exp \left(-{E}_2/\left({k}_BT\right)\right)\right], $$

Where *I*(*T*) is the integrated PL intensity and at temperature, *T*; *k*_B_, α, *C*_1_, and *C*_2_ are constants; and *E*_1_ and *E*_2_ are the thermal activation energies [25, 26]. The PL emission in the low-temperature range is mainly determined by *C*_1_exp(−*E*_1_/(*k*_*B*_*T*)) with *E*_1_ = 4.1 meV for SQDs and 14.5 meV for BQDs. The activation energies extracted from PL emission in the high-temperature range are *E*_2_ = 21.2 meV for the SQDs and 79.0 meV for the BQDs, which are generally understood to be due to thermally activated carriers escaping from the QDs. We attribute the smaller *E*_2_ for the SQDs to the relatively low energy of the surface states providing a lower energy channel for carrier escape.

The PL peak energies of the BQDs and SQDs also exhibit clear differences with increasing temperature, as shown in Fig. 4c, d, respectively. The PL peak energies of the BQDs show the well-known “S-shape” with a slow redshift at low temperature, then a fast red-shift through the middle range of temperatures followed by a relatively slow red-shift again as we approach room temperature. This feature can be attributed to carrier thermal activation and redistribution characteristics among BQDs, which correlate with the FWHM changes shown in Fig. 4e. Very differently, the SQD peak energy follows the Varshni law for the band gap of bulk InGaAs due to the absence of the carrier redistribution channel. This is also consistent with the monotonic increase of the FWHM of the SQDs throughout the entire temperature range as shown in Fig. 4f.

**Fig. 5:**
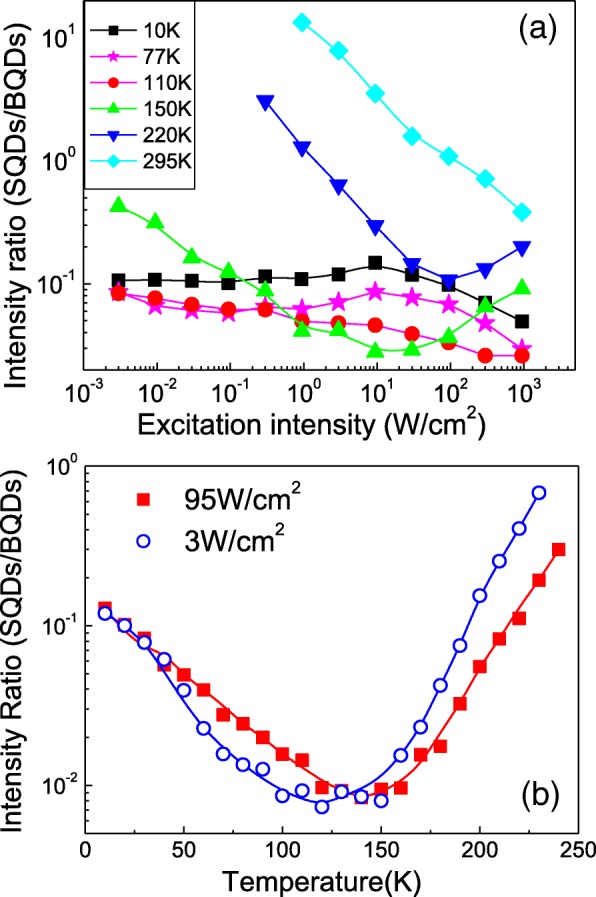
**a** The integrated PL intensity ratio (SQDs/BQDs) with respect to excitation intensity. **b** The integrated PL intensity ratio with respect to temperature for both low and high excitation intensity of 3 W/cm^2^ and 95 W/cm^2^

In addition to the nonradiative loss channels found through the temperature-dependent PL, it is evident from Fig. 4a that the decay rate with temperature of the SQDs also varies with excitation power, demonstrating that the carrier transfer rate is also excitation power dependent. The carrier population and corresponding PL intensities reflect the carrier transfer processes, thus the difference in these processes between the BQDs and SQDs can be characterized by the ratio between their PL intensities. So, we have plotted the ratio of the integrated PL intensities between the SQDs and BQDs as functions of excitation intensity and temperature in Fig. 5a, b, respectively.

As indicated by Fig. 5a, the ratios show different dependencies on the excitation intensity at different temperatures. At the low temperature of 10 K, the intensity ratio is much lower than 1 for all intensities, which is most likely due to the surface states acting as nonradiative recombination centers and competing with the SQDs to capture and “freeze” most of carriers. As the excitation laser intensity increases from 3 mW/cm^2^ to 950 W/cm^2^, the ratio first very slightly increases with a maximum around 10 W/cm^2^. This is a very slight effect likely demonstrating that there is some interrelation between the two systems. Here, the BQDs likely show some saturation which enhances the SQD emission. This can be seen in Fig. 3b where the BQDs have a slight deviation below the linear increase with power and the SQDs have a slight deviation above linear. At 77 K, the ratios follow nearly the same trend as that for 10 K, except that at 110 K, the ratio shows a monotonous decrease with laser excitation over the entire range. This likely shows the beginning of the population increase of the excited states of the BQDs which would have a greater than linear power law. This continues on in the 150 K data which can be compared with Fig. 3d, where the BQDs can be seen to increase at a rate slightly above linear, while the SQDs remain linear. Therefore, the data for 150 K in Fig. 5a shows a very noticeable decay in the ratio with increasing power. However, above ~ 10 W/cm^2^, the relationship changes direction apparently where the SQDs start to fill excited states with a greater than linear increase with power. This can possibly be seen in Fig. 4f where just above 100 K, the FWHM sharply increases, likely due to excited states being thermally populated. For higher temperatures in Fig. 5a, the ratio continues to follow the trend which is set at 150 K, with a continuous shift to higher values as the BQDs show increased signs of thermal quenching seen in Fig. 4a.

Figure 5b shows the change of ratio with temperature, decreasing first, then increasing for both low- and high-power excitations of 3 W/cm^2^ and 95 W/cm^2^, respectively. This can be understood completely by re-examining Fig. 4a. We see the BQDs to be stable up to ~ 150 K while the SQDs decay, then the BQDs suddenly decay with the SQDs continuing to decay slowly at a similar rate to the low temperature range. Thus, the ratio is influenced mainly by the sudden thermal quench of the BQDs over the background of the slow thermal loss of the SQD carriers to the surface states.

## Conclusions

In conclusion, we have carefully investigated the optical properties of self-assembled InGaAs/GaAs SQDs in composite nanostructures with the InGaAs SQDs separated from an InGaAs BQD layer by a thick GaAs spacer, but with varied QD areal density controlled by using different growth temperatures. Such SQDs behave differently from the BQDs, depending on the SQD surface morphology. For the best SQD and BQD sample in this study, the excitation intensity-dependent PL measurements show that the carrier emission efficiency is small at low temperature in comparison with the BQDs, but becomes relatively larger at room temperature as the BQDs go through a thermal quenching. Additionally, the integrated PL intensity and FWHM of the SQDs show monotonically decreasing and increasing dependencies on temperature, respectively. Finally, the ratio of the integrated PL intensity between the SQDs and BQDs show different changes with temperature and excitation intensities. These abnormal PL characteristics of the SQDs suggest a strong interaction and carrier transfer between the SQDs and surface states, depending not only on surface morphology but also on temperature and excitation.

## Abbreviations

AFM: Atomic force microscopy
BQDs: Buried quantum dots
FWHM: Full width at half maximum
MBE: Molecular beam epitaxy
PL: Photoluminescence
QD: Quantum dot
SQDs: Surface quantum dots
